# Preoperative risk assessment for ambulatory sinonasal surgery

**DOI:** 10.1007/s00405-020-06435-4

**Published:** 2020-10-22

**Authors:** Hans Rudolf Briner, Andreas Leunig, Christoph Schlegel, Daniel Simmen

**Affiliations:** 1 Center for Otorhinolaryngology, Head and Neck Surgery, Klinik Hirslanden, Witellikerstrasse 40, 8032 Zurich, Switzerland; 2 Rhinology Center Munich and ENT-Clinic Dr. Gaertner GmbH, Possartstr. 27-31, 81679 Munich, Germany; 3 Department of Otorhinolaryngology, Head and Neck Surgery, Luzerner Kantonsspital, 6000 Lucerne 16, Switzerland

**Keywords:** Ambulatory sinonasal surgery, Preoperative risk assessment, Day surgery risk score

## Abstract

**Objectives:**

An increasing proportion of patients who are candidates for endoscopic sinus surgery can be treated as an outpatient. A preoperative risk assessment is needed to evaluate eligibility for day surgery. This study analyses the effectiveness of a risk assessment scoring system which examines medical, procedure-related, and socioeconomic factors.

**Design:**

Prospective multicenter study.

**Setting:**

Three center study including Klinik Hirslanden, Zurich, Switzerland, Luzerner Kantonsspital, Lucerne, Switzerland and HNO-Klinik München-Bogenhausen, Munich, Germany.

**Participants:**

Patients with endoscopic sinus procedures between January 1st, 2017 and December 31st, 2018.

**Main outcome measures:**

The “day surgery risk score” consisted of three subgroups with medical, procedure-related and socioeconomic risk factors were assessed to determine if these predicted the severity of postoperative complications.

**Results:**

Three-hundred and one patients who underwent endoscopic sinus surgery were included. The score resulted in a median value of 5 [5, 5]. In the Receiver-Operating Curve (ROC—the true-positive rate against the false-positive rate), the Area Under the Curve (AUC) was 0.59 with 95% confidence interval from 0.49 to 0.69, indicating that the “day surgery risk score” may be no better at predicting the likelihood of a complication than a random classification model.

**Conclusions:**

The “day surgery risk score” is a straightforward risk assessment which combines medical, procedure-related, and socioeconomic factors. The score is easy to use but in trying to decide whether a patient is eligible for ambulatory endoscopic sinus surgery it did not predict whether a complication was more likely to occur.

## Introduction

Endoscopic sinus surgery (ESS) for patients with chronic rhinosinusitis (CRS) is primarily reserved for those who are refractory to medical management [1]. It is a common procedure and responsible for significant health care costs. Due to advances in surgical technique, and the management of postoperative sequelae such as pain, bleeding and general discomfort their affect has been reduced [2]. It is possible to perform ESS on an increasing proportion of patients as ambulatory surgery [3–5]. To minimize cost, health care providers have a strong incentive to make ESS an ambulatory procedure. However, not all patients are suitable for day surgery. Patients with an increased risk of postoperative complications should remain in hospital until they are stable enough to be discharged from.

Several factors are known to increase the risk of postoperative complications and early readmissions to hospital. These include individual medical risk factors, those that relate to the extent and complexity of the procedure, and socioeconomic factors. Known medical conditions that lead to an increased risk of complications and readmissions are significant cardiac, respiratory and bleeding disorders [6, 7]. Patients with a high comorbidity score according to the American Society of Anaesthesiology (ASA) system of III and IV (or higher) are usually not regarded as eligible for day surgery (Table 1) [8–10]. The main procedure-related factor which increases the risk for day surgery is the length of the procedure [2, 10]. Complex and extensive endoscopic procedures inherently have a higher risk of major arterial bleeding or a cerebrospinal fluid (CSF) leak as well as revision surgery and these are contraindications for outpatient care [11]. Socioeconomic factors which must be considered are the patient’s accessibility to medical care and the presence of a responsible adult to care for them if their condition requires this [3].

**Table 1:** The American Society of Anesthesiologists classification of physical status (ASA PS) as revised in 2014

ASA physical status classification	Definition	Examples
ASA I	A normal, healthy patient	Healthy, non-smoking
ASA II	A patient with mild systemic disease	Smoker, well-controlled diabetes mellitus or hypertension, mild lung disease
ASA III	A patient with severe systemic disease	Poorly controlled diabetes mellitus or hypertension, chronic obstructive pulmonary disease, history of myocardial infarction (> 3 months)
ASA IV	A patient with severe systemic disease that is a constant threat to life	Recent myocardial infarction (< 3 months), ongoing cardiac ischemia, severe valve dysfunction, sepsis
ASA V	A moribund patient who is not expected to survive without an operation	Massive trauma, intracranial bleed with mass effect
ASA VI	A declared brain-dead patient whose organs are being removed for donor purposes	

There are several publications which analyze risk factors for ambulatory sinus surgery and it is recognized that the factors mentioned above, especially the patient’s medical comorbidities (ASA PS score), should be considered in the preoperative evaluation [2, 3, 5–7, 9–11]. However, no validated preoperative risk assessment algorithms for ambulatory sinus surgery have been published so far.

A comprehensive preoperative risk evaluation for patients with CRS and in whom ESS is planned can help to decide whether they are eligible for outpatient surgery. This study aims to analyze a risk evaluation with a simple score which includes medical, procedure-related, and socioeconomic factors; the “day surgery risk score”.

## Material and methods

### Ethical considerations

The study was approved by the Ethics Committee of the Canton Zurich, Switzerland.

### Study design and patients

In a prospective multicentric interventional study, patients where endoscopic sinus procedures were planned at the Center for Otorhinolaryngology, Head and Neck Surgery, Klinik Hirslanden, Zurich, the Department of Otolaryngology, Head & Neck Surgery, Luzerner Kantonsspital, Lucerne and HNO-Klinik München-Bogenhausen, Munich, in the period between January 1st 2017 and December 31st 2018 were evaluated. Due to the structure of the health care system in these centers, patients were admitted with a minimal hospital stay of 24 hours. Patients below the age of 18 were excluded. Patient specific data (patient age, gender), the type of the procedure, the preoperative risk score or “day surgery risk score”, postoperative complications during the first 14 days and the length of hospital stay were analyzed. Data collection was performed using “ENTstatistics” software (ENTstatistics, Innoforce, LI-9491 Ruggel, Liechtenstein).

### Preoperative risk evaluation— “day surgery risk score”

The preoperative risk evaluation was performed that included the patient’s medical conditions, factors related to the planned endoscopic procedure, and socioeconomic factors.

The subgroup based on their medical factors was determined using the comorbidity score according to the American Society of Anaesthesiology (Table 1) [8]. A score of 1 meant that the patient was healthy. A score of two was given to patients with mild systemic disease without substantive morbidity, for example, controlled hypertension and mild lung disease and also to smokers. A score of three included patients with severe systemic disease and substantial morbidity such as poorly controlled hypertension, chronic obstructive pulmonary disease or a history of myocardial infarction (> 3 months). A score of four was reserved for patients with severe systemic disease that is a significant threat to life for example a recent cardiac infarction (< 3 months), ongoing cardiac ischemia or severe valve dysfunction.

Procedure-related risks were also rated within a score between 1 (minimal surgical risk) and 4 (high surgical risk). A score of one was given for an infundibulotomy and partial anterior ethmoidectomy, a score of 2 for anterior and posterior ethmoidectomy. A score of three was given if the procedure included the frontal and the sphenoid sinuses (frontoethmoidectomy, sphenoidectomy) and a score of four was defined for extensive endoscopic procedures such as tumor surgery or surgery of the anterior skull base.

The socioeconomic factors were also scored from 1 to 4. A score of 1 was given to patients who were independent with good access to medical care, whereas a score of two was given to patients which relied on help but otherwise had good access to health care. A score of three was given to patients who were independent but had limited access to health care, for example if they live far away from health care providers in the postoperative period. The score of four was reserved for patients who relied on help and had limited access to health care.

Each of these three groups were, therefore, given a score between 1 (low risk) and 4 (high risk). The addition of these three groups scores results in a total “day surgery risk score” between 3 and 12 (Table 2).

**Table 2:** Preoperative risk evaluation for endoscopic sinus surgery— “day surgery risk score”

Score	1 minimal risk	2 low risk	3 moderate risk	4 high risk	Total	
Medical condition (ASA I-IV)^1^	Healthy	Mild systemic disease	Severe systemic disease	Severe systemic disease that is a constant threat to live		
Surgical procedure	Infundibulotomy, partial anterior ethmoidectomy	Anterior and posterior ethmoidectomy	Frontoethmoidectomy, sphenoidectomy	Extensive tumor surgery, anterior skull base surgery		
Socioeconomic factors	Independent patient, good access to health care system	Patient needs support	Limited access to health care system	Patient needs support, limited access to health care system		
Total (3–12)						

^1^Comorbidity score according to the American Society of Anaesthesiology *ASA*, see also Table 1

It is important to realize, that the three scores of each subgroup made up the “day surgery risk score” with each representing different postoperative “areas of risk”. It is established in the literature, that the individual medical risk factors (ASA PS score) correlate with the frequency and severity of postoperative complications [8–10]. The same is true for the procedure-related factors [2]. In contrary to these two factors, the socioeconomic factors do not have a direct impact to the rate and severity of postoperative complications. However, in the rare case where a complication occurs, the socioeconomic factors are important because they determine the ability to access fast and adequate medical care to treat any complication and this may, therefore, have an impact on the overall outcome.

### Assessment of postoperative complications

The most common postoperative complications after ESS are bleeding and pain [12, 13]. The postoperative complications were graded according to their severity from 1 (none, very mild) to 4 (severe). Grade 1 included patients with no or only very mild complications which could be controlled without the need for additional care. Grade 2 included patients with mild complications, for example minor bleeding or pain which could be managed by conservative actions and did not require hospital care. Grade 3 contained patients with moderate complications such as moderate bleeding or pain requiring specific therapy during hospitalization or readmission to hospital. Grade 4 involved patients with severe postoperative complications such as marked bleeding or pain requiring hospital care or urgent revision surgery. In practical terms, only grade 3 and grade 4 incidents were regarded as “complications” for our analysis, because of their need for hospital care.

### Empirical evaluation

Statistical analysis was only conducted to evaluate discriminatory properties of the proposed score based on the empirical data provided.

Descriptive statistics of patients’ baseline characteristics were presented as mean and standard deviation (SD) for continuous measurements. Categorical variables, as well as the scores were summarized as number and percentage of total or as median and interquartile range (IQR). Mosaic and bar plots were used to summarize the proportion of complications within the risk score categories. A Receiver-Operating Curve (ROC) visualizes the true-positive rate (TPR) against the false-positive rate (FPR) at all thresholds along the risk score (3–11). The area under the ROC curve was computed and reported with 95% confidence intervals. All analyses were conducted with R (version 3.6.2, 1) [14].

## Results

A total of 301 patients (174 males, 127 females) with a mean age of 51.2 years (14.8) were included in the study. The median length of hospitalization was 2 [IQR 2, 2] days.

224 of the patients had a preoperative risk score for the patient’s medical condition of 1, indicating that most of the patients were healthy or had minimal medical risk factors. Most patients had a low or moderate risk from their surgical procedure with 77 patients having a score of 1 or 2 and 214 patients a surgical score of 3. Eighty-four percent of the patients had a socioeconomic score of 1, indicating that a majority of patients were independent and had good access to the health care system. No patient had the maximal socioeconomic score of 4. The sum of the three scores from each category resulted in the “day surgery risk score” with a median value of five [5] and ranged from a score of 3–11.

Complications requiring urgent therapy during the hospitalization, or with the need of readmission occurred in 46 patients; 39 patients with grade 3—moderate degree of complications, and seven patients with grade 4—severe complications. Most of these patients suffered from pain or bleeding. Other complications included nausea and a moderate malaise in eight patients, orbital emphysema without need for surgical intervention in five patients and panic attacks in two patients. All complications could be treated with medication and any bleeding was controlled by packing. There was no need for immediate revision surgery and there were no permanent sequelae. In the seven patients with grade 4, the complications could be controlled during their primary admission in five of them but in two of them a readmission was necessary (postoperative day 6 and 9). Both of these patients had a day surgery risk score of 6, one of them suffered from an exacerbation of pain, the other had bleeding which was controlled by packing.

**Fig. 1:**
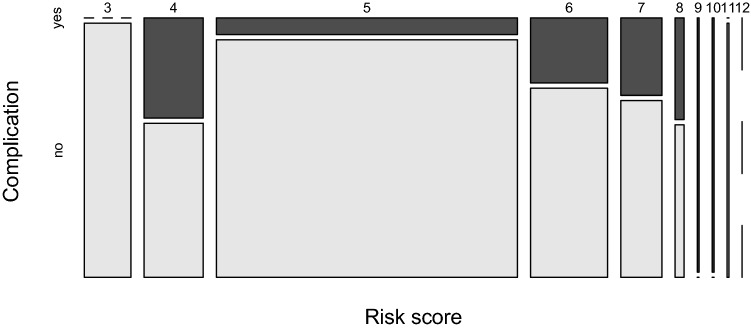
Frequency of complications along the range of the risk scores with the area of each tile being proportional to the number of patients within the risk score category

**Fig. 2:**
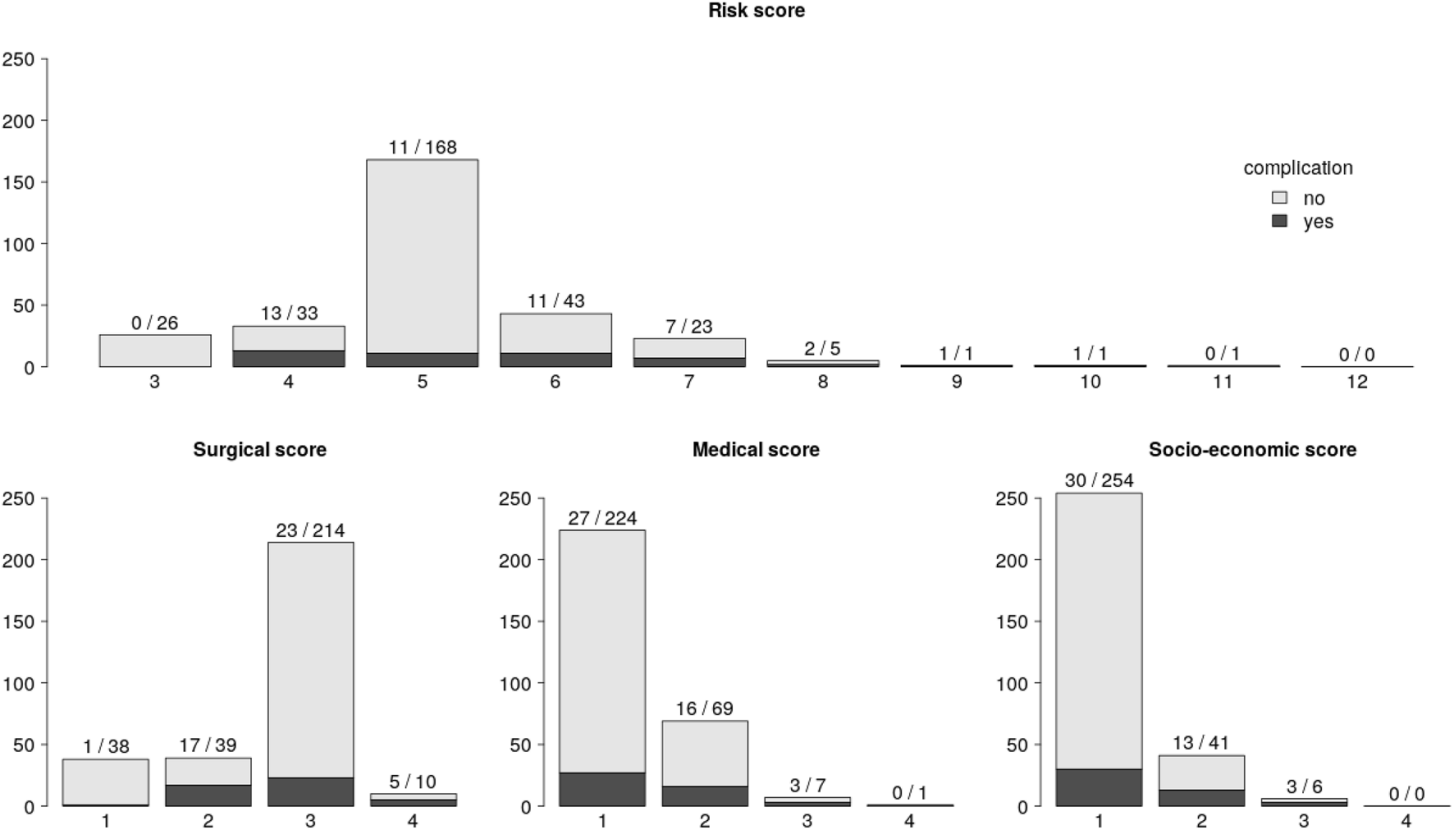
Bar plots of total number of complications along the total and sub-risk scores

The relationship of the day surgery risk score to complications is summarized in (Figs. 1, 2).

**Fig. 3:**
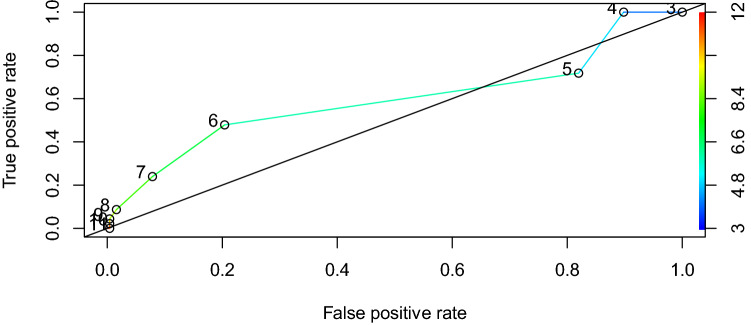
Receiver-Operating Curve (ROC) illustrating the true-positive rate (TPR) against the false-positive rate (FPR) at all thresholds of the risk score. The black diagonal line indicates a reference curve with an AUC of 0.5

The Receiver-Operating Curve (ROC) in Fig. 3 assesses the true-positive rate (TPR) against the false-positive rate (FPR) at all thresholds of the “day surgery risk score”. The Area Under the Curve (AUC) was 0.59 with 95% confidence interval from 0.49 to 0.69 measuring the capability of distinguishing between the cases (complications yes or no). The lower confidence interval of the AUC of the risk score was smaller than the reference value of 0.5, indicating that the risk score may be no better at predicting complications than a random classification model. ROC analysis of the individual subscores revealed that the surgical risk score does not have better predicting properties with an AUC of 0.48 (0.40–0.57). The medical risk score (ASA) alone with an AUC of 0.6 (0.52–0.67) was only marginally in being able to discriminate.

## Discussion

Endoscopic sinus surgery is a common procedure for patients with CRS refractory to medical management [1]. Due to advances in surgical technique, postoperative complications such as bleeding and pain can usually be minimized. This allows ESS to be performed as an ambulatory procedure in an increasing proportion of patients [2–7, 9–11]. However, despite the advances in surgical technique, ESS may still lead to potentially life-threatening complications [13]. Patients with an increased risk of postoperative complications should remain in hospital until they are stable enough to be discharged from hospital care to avoid urgent hospital readmission and a potentially dangerous situation [12].

Preoperative risk evaluation is key in evaluating whether a patient is eligible for ambulatory surgery or not. There are known risk factors which increase the probability of postoperative complications and early readmissions to hospital. These are individual medical risk factors and factors which relate to the extent and complexity of the procedure [2, 6, 7, 9–11]. There are also socioeconomic factors which may have an impact of the postoperative outcome, because they may impair the ability to access treatment in a timely fashion if a complication occurs [3].

There are several published guidelines for preoperative risk evaluation where ambulatory ESS is being considered [3, 7, 9]. These recommendations are based on known risk factors, mainly medical factors such as the ASA comorbidity score and bleeding disorders, but also the extent and complexity of the procedure and socioeconomic factors. However, due to different health care systems in different countries, there are no universally accepted guidelines for the preoperative risk assessment for ambulatory ESS patients.

The “day surgery risk score” evaluated in this paper offers a comprehensive preoperative evaluation by adding three scores containing medical, procedure-related and socioeconomic risks to one overall score. Each of these scores ranges from 1 (minimal risk) to 4 (high risk), which makes it easy to apply. The overall “day surgery risk score” ranges from 3 to 12, and the hypothesis was that this reflects an increasing individual preoperative risk. However, in the cohort of 301 patients assessed in this paper, statistical analysis revealed that the “day surgery risk score” may not be better in predicting complications than the ASA score alone or even a random classification model. There are several factors which must be considered in the discussion of this “negative” result. In our cohort, the distribution of the risk scores was unequal. A majority of 214 patients had a surgical score of 3 whereas only 39 patients had a surgical score of 2. Looking at the total risk score, only 31 of the 301 patients had a score of 7 or higher and there was no patient with the maximum score of 12. This unequal distribution may be a confounding factor although statistical analysis could not confirm this in our cohort. Another variable which might influence the rate of complications is the surgeon’s experience. All patients included in this study were operated by experienced rhinosurgeons. This could be a factor which leads to lower complication rate, especially in more extensive surgical cases. Furthermore, this negative result must be regarded in the context that the “day surgery risk score” is a compound score including socioeconomic risk factors which have no correlation with the frequency of postoperative complications. The socioeconomic factors may increase the risk that a patient may not receive fast or adequate treatment and have an adverse affect on the overall outcome. The design of the present study looked solely at the frequency and severity of complications and did not allow for a more global evaluation.

An interesting finding was the fact, that the surgical risk subscore did not predict complications any better in our cohort than a random model. A possible explanation for this finding could be the unequal distribution of risk scores as mentioned above and that all patients were operated on by experienced surgeons which would lead to an expected low complication rate. Even the medical risk factors subscore (ASA PS) alone was not good in predicting a complication with an AUC in the ROC of 0.6 (0.52–0.67). This indicates that the surgical and the medical risk factors alone have a limited value in predicting the risk of postoperative complications. This finding correlates with the clinical experience that the occurrence of a complication after ESS also depend on multiple and sometimes unpredictable factors other than the factors mentioned above.

The final decision whether ambulatory ESS is advisable may depend not only on the preoperative risk factors that are summarized in the “day surgery risk score”, but other factors such as the relevant national health care system, culture and the local health infrastructure. The preoperative risk evaluation with the “day surgery risk score” did not prove to be a good predictor of postoperative complications. The addition of the scores for the patients’ medical status, the extent of surgery and their socioeconomic situation alone may be too simplistic to help determine a patient’s suitability for outpatient surgery.

### Limitations

The day surgery risk score attempted to assess preoperative risk and suitability for outpatient surgery. However, the results in the cohort of 301 patients analyzed in this paper revealed that the score may not be better in predicting complications than a random classification model. Further studies are necessary using other variables or methods of analyzing them to predict whether a patient can be managed as an outpatient.

## Conclusion

The “day surgery risk score” attempted to provide a comprehensive risk assessment for a patient’s suitability for outpatient surgery and it includes medical, procedure-related and socioeconomic factors. The score is easy to use but it did not predict whether a complication was more likely to occur.

## Data Availability

Not applicable.
