# Correction to: Preoperative risk assessment for ambulatory sinonasal surgery

**DOI:** 10.1007/s00405-020-06475-w

**Published:** 2021-02-03

**Authors:** Hans Rudolf Briner, Andreas Leunig, Christoph Schlegel, Daniel Simmen

**Affiliations:** 1 Center for Otorhinolaryngology, Head and Neck Surgery, Klinik Hirslanden, Witellikerstrasse 40, 8032 Zurich, Switzerland; 2 Rhinology Center Munich and ENT-Clinic Dr. Gaertner GmbH, Possartstr. 27-31, 81679 Munich, Germany; 3 Department of Otorhinolaryngology, Head and Neck Surgery, Luzerner Kantonsspital, 6000 Lucerne 16, Switzerland

## Correction to: European Archives of Oto-Rhino-Laryngology 10.1007/s00405-020-06435-4

In the original publication of the article, under the abstract section, the subsections “Ethical consideration” and “Study design and patients” was included incorrectly. The correct abstract includes the subsections Objectives, Design, Setting, Participants, Main outcome measures, Results and Conclusions.

In addition, under the material and methods section, the subsections “Ethical consideration” and “Study design and patients” was published incorrectly. The correct text should read as below,

**Material and methods**

**Ethical considerations**

The study was approved by the Ethics Committee of the Canton Zurich, Switzerland.

**Study design and patients**

In a prospective multicentric interventional study, patients where endoscopic sinus procedures were planned at the Center for Otorhinolaryngology, Head and Neck Surgery, Klinik Hirslanden, Zurich, the Department of Otolaryngology, Head and Neck Surgery, Luzerner Kantonsspital, Lucerne and HNO-Klinik München-Bogenhausen, Munich, in the period between January 1st 2017 and December 31st 2018 were evaluated. Due to the structure of the health care system in these centers, patients were admitted with a minimal hospital stay of 24 h. Patients below the age of 18 were excluded. Patient specific data (patient age, gender), the type of the procedure, the preoperative risk score or “day surgery risk score”, postoperative complications during the first 14 days and the length of hospital stay were analyzed. Data collection was performed using “ENTstatistics” software (ENTstatistics, Innoforce, LI-9491 Ruggel, Liechtenstein).

The original article has been updated.

